# Pandemics, populism and bioethics: A critical approach

**DOI:** 10.1371/journal.pgph.0004576

**Published:** 2025-05-20

**Authors:** Gabriela Arguedas-Ramírez, Ewen Speed, Zackary D. Berger, Russell Mannion

**Affiliations:** 1 School of Philosophy, University of Costa Rica, San José, Costa Rica; 2 School of Health and Social Care, University of Essex, Colchester, United Kingdom; 3 Division of General Internal Medicine, Johns Hopkins School of Medicine, Core Faculty, Berman Institute of Bioethics, The Johns Hopkins Outpatient Center, Baltimore, Maryland, United States of America; 4 Health Services Management Centre, University of Birmingham, Birmingham, United Kingdom; UNAM: Universidad Nacional Autonoma de Mexico, MEXICO

## Introduction

The rise of populist leaders and movements around the globe is one of the most significant political developments of the 21^st^ Century with profound implications for public health. Here we consider the implications for bioethics and public health ethics from right-wing political populist responses to the COVID-19 pandemic. A key issue raised by many of these responses is the lack of critical engagement by mainstream bioethical approaches with contemporary political developments such as the growing influence populist politics. We address this question by exploring how bioethics can better engage with insights and approaches drawn from the field of political science [1,2].

There are many competing definitions of populism [3–6], but most view populism as a political position that differentiates a binary moral classification between ‘the people’ as a morally, virtuous group, contrasted against a corrupt ‘elite’, characterised as a small illegitimate group who do not represent the will of the people. Populist political movements operate in this space, claiming to represent ‘the people’ against the elite [2]. Populist politics can sit anywhere along the political spectrum. Right-wing populism tends to combine anti-elite rhetoric with other polarizing and discriminatory narratives that exploit antipathies based on cultural and national identity, race, and gender, amongst other social categories. Left-wing populism tends towards championing the ‘will of the people’ against an economically privileged elite. The key distinction between left and right-wing populism is in the enemy they identify. Right-wing political populism is now in the ascendancy across the globe (even more so after Donald Trump’s re-election and the reassertion of Make America Great Again (MAGA) politics), but left-wing populism has also been evident in different ways [7].

In this paper we focus on identifying the negative impact right-wing populism had on public health policy during the COVID-19 pandemic. We argue that if specialists in public health ethics and bioethics are not better prepared to understand the ways in which anti-democratic and populist leadership can co-opt and instrumentalize these fields, it is likely they will face considerable obstacles in fulfilling their role under such political conditions. Our premise is that many right-wing politicians and actors utilised a political response to the pandemic as a means of imbricating right-wing policies into mainstream public health discourse. The most recent and alarming case that illustrates this point is President Trump’s decision to appoint Robert Kennedy Jr as the lead in the Department of Health and Human Services. Kennedy’s alignment with right wing populist tropes, already evident for years, became more apparent with his prominent anti-vaccination stance as well as his efforts to minimize the threats of the COVID-19 pandemic.

Many of these appeals to common right-wing populist political tropes, such as health and social care nativism, welfare chauvinism and the arrogance of ignorance [8], run contra to any effective global public health response to the pandemic. Many right-wing populist political leaders have been most vocal around COVID-19, polarizing public discussion of the issues, as well as promoting vaccine hesitancy, undermining the role of medical expertise, validating conspiracy theories and promoting anti-scientific narratives [9].

## Populism and COVID-19

Whilst populism can be found at all points on the political spectrum, this paper focuses on right-wing populist responses to COVID-19. There are numerous explanations for the upsurge in populism [10] which reflect current modes of global capitalism. There is a burgeoning literature on the global rise of populist politics in relation to health and social care [11–14]. The COVID-19 pandemic has presented both barriers and opportunities to right-wing populist politicians. The need for an effective response to COVID-19 created a notable paradox for populist positions, whereby populist claims to speak on behalf of ‘the people’ are conflated with an opposition towards effective population level health interventions which would improve population mortality and morbidity in the face of the pandemic, such as opposition to social distancing, masks and vaccination programmes. A fundamental reason why many right-wing populist leaders opposed those public health interventions was economic. They deployed false narratives against the WHO recommendations in order to convince the majority of the people to reject public health measures that are costly for the economy (and therefore require redistributive justice) but were simultaneously necessary to effectively control the pandemic and prevent overwhelming hospitals. Trump took this confrontation with the WHO to the extreme level of giving the order to withdraw the US from the WHO, causing a global health turmoil that will have extremely damaging consequences worldwide.

Moreover, right-wing populism tends to be committed to neoliberal economic policies that problematise or even oppose public health interventions that require public financing and state intervention in the business sector and the economy. The most effective public health interventions during the pandemic required significant state intervention and regulation of private activities. This was something that right-wing politicians were keen to avoid, due to the cost and the potential negative impact on the economy [15]. To effectively counter this reaction to pandemics and to preserve the integrity of the public health response, there is a clear need to move beyond individualistic behavioural explanations and consider the political context of science denial - in particular the overlap between right wing populism and anti-science movements [16]. In short there is a need for a new form of politically inflected bioethics.

Populist leaders portray a political context in which they—and they alone—represent the true voice of the people. In the context of the COVID-19 response, these anti-elitist populist sentiments have typically targeted technocratic elites, who are viewed as representing the establishment and therefore as operating against the will of the people. This includes scholarly institutions, scientific experts, medical authorities, and big corporations [17–19]. Populism valorises ‘folk wisdom’ and ‘common sense’ over scientific expertise [20]. Similarly, anti-science rhetoric has a long association with conspiracy theories [21–24]. In the debate around COVID-19 vaccination, the initial right-wing response was mobilised around well-rehearsed ideological tropes. Key concerns around COVID-19 vaccine hesitancy tend to concern questions of scientific expertise and freedom of choice [25].

Although government responses to the pandemic differed, there were clear similarities across right-wing populist political responses, which we argue coalesce around four common themes: first, initial denial and then poor management of the pandemic. Second, concerted efforts to frame the pandemic as an economic rather than a public health crisis; third, (relatedly) a questioning of scientific and professional expertise. Lastly, comes a process of ‘othering’ of marginal groups for political ends [26]. In this context, the response to COVID-19 vaccination was complex. Much of the populist reaction to vaccines was based on previous populist responses to other mitigation measures in the context of the pandemic [27]. There were already well-rehearsed right-wing responses (typically couched in libertarian rhetoric, e.g., the so-called Barrington declaration, [28]) which raised concerns about the extension of state surveillance into previously protected areas of civil society and claims about the erosion of civil liberties (e.g., in debates about mask-wearing, or so-called ‘muzzling’) [29]. From testing to vaccines to boosters, the politics of scientific practices and pronouncements have been front and centre from the very beginning of the pandemic. However, the USA federal Food and Drug Administration (FDA), despite being privy to the latest science in February 2020, systematically failed to distribute functional rapid testing for COVID-19, and refused to introduce other tests from other countries on the grounds of scientific incompatibility, suggesting that politically, public health decisions tend to be accomplished in the context of political expediency in response to a crisis rather than any broader engagement with social democratic (or public health) practice [30]. This has serious implications for all forms of public health policy and practice.

Consider that many low and middle income countries (LMIC) depend on the FDA and EMA to decide which criteria to use in the process of evaluation and approval of vaccines. Given that the pharmaceutical companies that produce those vaccines approved in the US and EU, failed to quickly scale up production to meet the global demand, coupled to the fact that the COVAX mechanism did not secure enough vaccines for LMIC [31], many countries established bilateral or regional negotiations with pharma companies and HIC [32]. However, many LMIC have accused US-based pharma companies of predatory terms and practices [33]. The bioethical implications of these agreements are doubly disturbing, in that there is evidence that private profit took precedence over global health [34,35].

Clearly, public health imperatives did not drive these programs. There are several social political and economic factors at play, which overlapped and combined to reduce the efficacy of a global vaccine rollout. Internationally, Russia and China took the lead in attempts to dominate the vaccine market in Latin American and African countries [36] - EU countries and the US missed out on this emerging market as they did not initially support COVAX. Indeed, it was not until June 2021 that the US changed its policy regarding international cooperation and humanitarian aid to tackle this pandemic [37]. As the pandemic played out, it appears that decision making processes at the highest levels of power were based on geopolitical and economic considerations, leaving global health justice and global bioethics outside the door. At the same time, distrust and lack of transparency in the negotiations between governments, international organizations and the private sector might become fuel for social mobilizations that end up being co-opted by populist political leaders. It is precisely these concerns which led to allegations of corruption against the Brazilian government around the purchase of vaccines [38].

## Bioethics as a politically contested field

We consider bioethics as a field of political contestation. Furthermore, we contend that claiming that bioethics is (and indeed should remain) “apolitical” is implicitly adopting a political position that lends support to maintaining and reproducing the status quo, which, it appears is swinging towards anti-scientific right-wing populism. Advocates for political engagement in the field of bioethics [39,40] view bioethics as being influenced by different political levels, which delimit its contours and influence what does (and does not) fall within its purview. Currently mainstream approaches to bioethics tend to be overly dependent on frameworks and methodologies that isolate its analysis from wider political and social contexts [40].

For example, pre-COVID-19, there were warnings about the implications for science and public health policy of President Trump’s decision to prevent staff in the US Centre for Disease Control (CDC) from using seven words (diversity, transgender, vulnerable, foetus, entitlement, evidence-based and science-based) [41]. Indeed, this tactic is being repeated with recent moves to halt all Diversity, Equity and Inclusion programmes in US federal offices [42]. Trump’s 2018 demands foreshadowed the approach that would be adopted by the Trump administration during the pandemic, which played to several right-wing ideology relating to reproductive health, sexual health and scientific expertise. Similar scenarios have played out in Latin American and European countries, where governments have attempted to control the range of theories and conceptual frameworks that could be taught in universities, [43–45].

There is overlap when we consider Bolsonaro’s populist response to the COVID-19 pandemic in Brazil. After the pandemic a national inquiry was established the country’s senate into the governments’ response, and it led to the indictment of federal authorities, government aides, and companies for crimes committed during the pandemic. Then President Bolsonaro was accused of making false statements, promoting fake treatments and, more importantly, “crimes of responsibility”, and some argued that the charge of crimes against humanity levelled against Bolsonaro makes a valuable contribution to global health and the future of global pandemic responses [46]. They are indeed an example of a politically informed bioethics. For example. the indictment found that the Brazilian governmental approach encouraged people to ignore the advice of epidemiologists and the scientific community.

There are abundant examples of how populist political agendas have sought to influence freedom of thought and action in several health policy contexts [26]. Bioethics is particularly vulnerable to these intrusions because of the nature of its subject matter. Characteristic themes in bioethics, such as life and death decision making processes, abortion, euthanasia, scarce resource allocation, and so forth, touch deeply emotional fibers in the general public. Bioethics encompasses fraught issues whose nature reflects rifts in the wider politics of health that are not often explicitly discussed. For instance, consider public debates about euthanasia/Medically Assisted Dying identifying which lives are worth living, the ethics of these types of decisions were often only implicit in much of the COVID-19 discourse [47].

To counter these intrusions, different forms of bioethics which are politically engaged are required, forms which are capable of being critical of political moves to debilitate democratic principles and institutions, such as reduced transparency, accountability and representativeness in health policy and practice. To accomplish this, it is necessary to reassess both what the field of bioethics encompasses and what conditions are required for bioethics to address these political challenges.

## A broader understanding of bioethics and global health

What is the role of bioethics, both actual and potential? What are the ethical duties of bioethicists? Do such duties differ among countries in the Global North and the Global South? Moreover, how relevant can bioethics be in relation to right-wing populist political attacks on public health policy and practice? To address these questions a crucial first step is that bioethics –broadly understood- should take a leading role in re-examining the relationship between science (including biomedicine) and society, specifically in terms of addressing what might be at stake (ethically) in terms of the prioritisation of one or other specific policy approaches.

This re-evaluation requires stronger, (and more explicit) links with academic disciplines such as science and technology studies, political philosophy, sociology of health, and public health ethics, amongst other fields. Bioethics should have a leading role not only in building trust between science, public policy and the people, but also in considering alternative structures that can ensure accountability, transparency and participation, based on social democratic principles [48]. Indeed, bioethics in driving public discussions around these issues should already be problematising processes of medicalization of public health and public health ethics. In addition, there should be an open dialogue regarding how bioethics in the Global South differs from mainstream bioethics in the Global North and how such differences can enrich the debate about the relationship between public health and bioethics.

The COVID-19 pandemic provides an opportunity for reimagining this role. Bioethics in the Global North evolved as a discipline in the context of biomedical research. It has continually struggled to bridge the individualistic focus of its earliest years with more recent developments which require a more intersectional understanding of the ethics of health care and health inequalities [49]. In this regard, there is a clear need for bioethics to engage directly with questions of democracy and social justice because the central issues in bioethics are influenced and shaped by how a society is organized and the nature and quality of its political institutions and discourse. In short, bioethics should take a normative position regarding politics. Recent developments around processes and the ethics of moral distress [50,51] might provide a suitable point to organise a response to policies which clearly breach principles of non-maleficence, beneficence, and justice, among others.

In this polarised political environment, truth claims can become symbolic of entrenched tribal identities. Experts are deemed wrong not only in terms of knowledge, but also in terms of their ideology, their morals and their underling motivations and ethics. This is a crucial component of the right-wing playbook. The populist revolt against expertise may be difficult to resolve unless one of the main drivers of populism – economic inequity - is addressed. A recent editorial published in The Lancet [52] brings attention to the powerful impact that misinformation and disinformation have on public health, emphasizing the need for health professionals to take responsibility on this issue and for scientific and medical communities to combat. Bioethics, as an academic and practical field of enquiry and action must take this call seriously as well, and the first step along this path is exploring the different ways in which right wing populist political leaders and movements use false information and lies to undermine the common good.

## Conclusion

Bioethics is a fundamental, normative field that underpins health-related disciplines. Therefore, it should be informed by the need to address inequality and social justice. In consequence, bioethics should have a role in opposing populist interventions in science and health-related fields that go against basic ethical principles of public health, especially under critical circumstances such as global pandemics. Moreover, bioethics should promote social participation, openness and inter-sectoral dialogue in science and health-related disciplines and services. In this way, societies can be better prepared to identify problematic behaviour by leaders which lead to ethically unacceptable decisions that endanger the population, such as policies which prioritise commercial and economic concerns over public health interventions.
